# 3D whole-heart noncontrast coronary MR angiography based on compressed SENSE technology: a comparative study of conventional SENSE sequence and coronary computed tomography angiography

**DOI:** 10.1186/s13244-023-01378-w

**Published:** 2023-02-15

**Authors:** Yang Zhang, Xinna Zhang, Yuqi Jiang, Panpan Yang, Xiankuo Hu, Bin Peng, Xiuzheng Yue, Yuanyuan Li, Peiqi Ma, Yushan Yuan, Yongqiang Yu, Bin Liu, Xiaohu Li

**Affiliations:** 1 Department of Radiology, The First Affiliated Hospital of Anhui Medical University, Research Center of Clinical Medical Imaging, Anhui Province Clinical Image Quality Control Center, Hefei, 230032 Anhui Province China; 2 Department of Radiology, Fuyang People’s Hospital, Fuyang, 236015 Anhui Province China; 3 Department of Radiology, Fuyang Hospital of Anhui Medical University, Fuyang, 236000 Anhui China; 4 Philips Healthcare, Beijing, China

**Keywords:** Acceleration factor, Compressed SENSE, Coronary angiography, Image quality, Magnetic resonance imaging

## Abstract

**Objective:**

The relatively long scan time has hampered the clinical use of whole-heart noncontrast coronary magnetic resonance angiography (NCMRA). The compressed sensitivity encoding (SENSE) technique, also known as the CS technique, has been found to improve scan times. This study aimed to identify the optimal CS acceleration factor for NCMRA.

**Methods:**

Thirty-six participants underwent four NCMRA sequences: three sequences using the CS technique with acceleration factors of 4, 5, and 6, and one sequence using the conventional SENSE technique with the acceleration factor of 2. Coronary computed tomography angiography (CCTA) was considered as a reference sequence. The acquisition times of the four NCMRA sequences were assessed. The correlation and agreement between the visible vessel lengths obtained via CCTA and NCMRA were also assessed. The image quality scores and contrast ratio (CR) of eight coronary artery segments from the four NCMRA sequences were quantitatively evaluated.

**Results:**

The mean acquisition time of the conventional SENSE was 343 s, while that of CS4, CS5, and CS6 was 269, 215, and 190 s, respectively. The visible vessel length from the CS4 sequence showed good correlation and agreement with CCTA. The image quality score and CR from the CS4 sequence were not statistically significantly different from those in the other groups (*p* > 0.05). Moreover, the image score and CR showed a decreasing trend with the increase in the CS factor.

**Conclusions:**

The CS technique could significantly shorten the acquisition time of NCMRA. The CS sequence with an acceleration factor of 4 was generally acceptable for NCMRA in clinical settings to balance the image quality and acquisition time.

## Key points

- The compressed SENSE could significantly shorten the scan time in NCMRA.
- The image quality tended to decrease with the decrease in scan time.
- The image quality tended to decrease with the decrease in scan time.
- The image quality of proximal vessels was higher than that of distal vessels.

## Introduction

Coronary artery disease (CAD) is one of the most frequent causes of morbidity and mortality globally [1]. Coronary X-ray angiography (CAG) and coronary computed tomography angiography (CCTA) are the most commonly used clinical imaging methods [2], but both of them have some limitations. The ionizing radiation harms patients, and some patients have contraindications such as iodine contrast allergy or renal failure. Several studies showed that many patients who underwent CAG did not have obstructive CAD. Hence, better strategies are required for risk stratification, informing decisions, and improving the diagnostic efficiency of cardiac catheterization in routine clinical practice, particularly in the young population, in patients who are contraindicated to iodinated contrast agents, and in patients requiring cardiac catheterization [3, 4].

Over the past decades, coronary magnetic resonance angiography (CMRA) has emerged as a noninvasive and radiation-free technique for assessing coronary artery direction, malformation, and proximal CAD [5–7]. A recent study demonstrated good diagnostic accuracy for CAD detection using high-resolution CMRA, with high sensitivity and negative predictive values [8]. Moreover, CMRA does not produce blooming artifacts from coronary calcification [9]. In previous studies, a free-breathing diaphragm-navigated steady-state free precession sequence was successfully applied in CMRA with the 1.5-T scanner, and a spoiled gradient-echo sequence was more commonly used at 3.0 T magnetic resonance for patients with suspected CAD [10–12].

Nevertheless, the limitations of CMRA in clinical practice include the long acquisition times and complex protocols for systematically optimizing the image quality for diagnosis. The patient’s heart rate, respiration rate, or body position may change during the long acquisition period, resulting in poor image quality or even unsuccessful imaging [13]. Some techniques for controlling the motion of patients have been used to reduce the limitations of CMRA, but its long acquisition time is still a major issue [14, 15].

The sensitivity encoding (SENSE), a parallel imaging technique that consists of functioning multiple coils as independent receivers, was described as a modified magnetic resonance imaging (MRI) technique for scanning various tissues and organs of the body [16–18]. However, the accelerating capability of parallel imaging is limited by the number of receiver coils, and the acceleration factor rarely goes beyond 4 in clinical setups due to concerns about potential imaging artifacts and signal-to-noise ratio (SNR) [19]. Recently, the compressed SENSE (CS) technique has been developed as a state-of-the-art reconstruction algorithm that allows the combination of a wavelet transformation of compressed sensing with the coil information of SENSE. Moreover, this algorithm has been used to accelerate MRI techniques, especially 3D acquisition [20, 21]. To date, many studies have used CS techniques for cardiovascular MRI, with CS acceleration factors ranging from 3 to 9 [22–25]. A CMRA study using compressed sensing technology showed that non-CMRA (NCMRA) with an acceleration factor of 9 could largely shorten the acquisition time. However, the image quality score and visible length were lower than those of conventional CMRA [26]. Another study comparing CS with the SENSE technique in vitro showed that, compared with SENSE, CS5 images had a significantly higher SNR and contrast-to-noise ratio (CNR). In contrast, CS2, CS3, and CS4 showed no advantage in terms of acquisition time, and CS6 yielded a significantly lower SNR [27]. Therefore, a more comprehensive study is required to verify the effectiveness of CS on NCMRA and to identify the optimal CS acceleration factor for routine clinical use.

This study aimed to evaluate the image quality of CS NCMRA using a 3-T scanner compared with conventional SENSE sequence and CCTA and identify the optimal CS acceleration factors for clinical use.

## Materials and methods

### Study participants

**Fig. 1:**
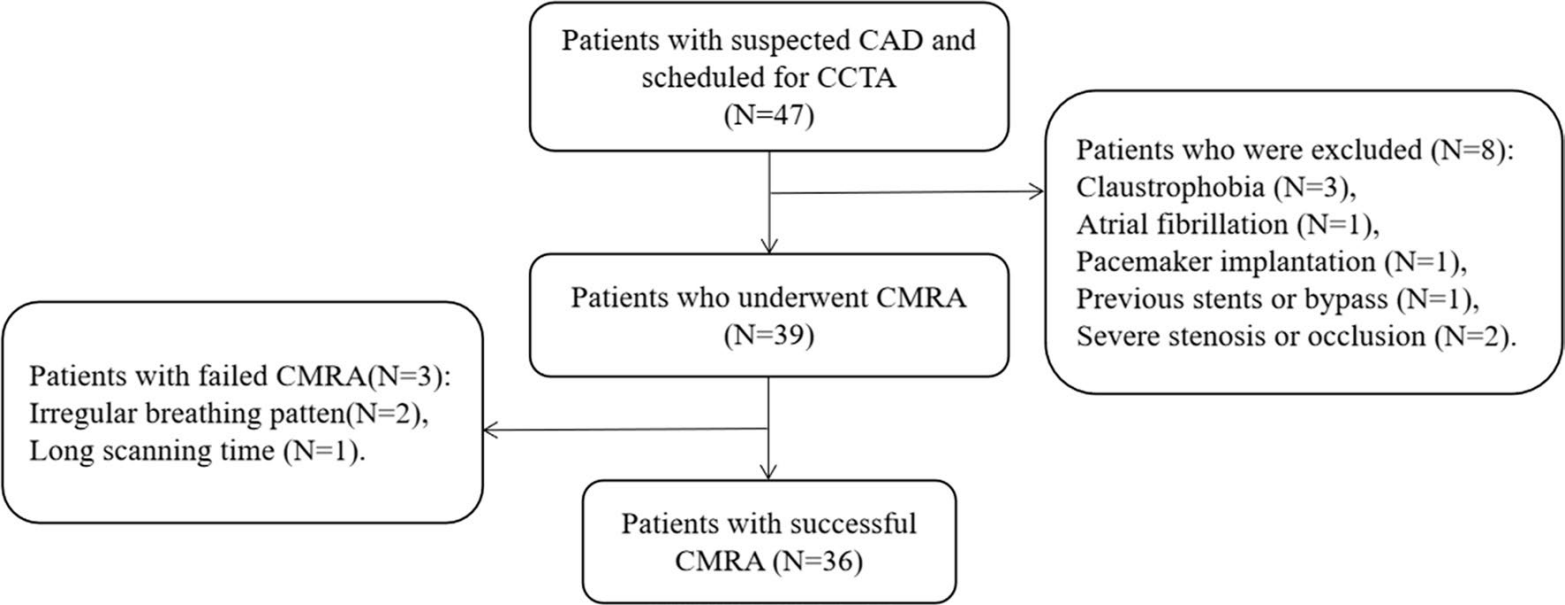
Flowchart of the study participants. CAD, coronary artery disease; CCTA, coronary CT angiography; CMRA, coronary MR angiography

Forty-seven patients with suspected CAD but without apparent coronary stenosis on CCTA were recruited for NCMRA examination at Fuyang People’s Hospital from September 2021 to March 2022. The exclusion criteria were severe arrhythmia, stenosis or occlusion, and MRI-related contraindications, including pacemaker implantation and claustrophobia. Our local ethics institutional review board approved this prospective study, and all participants provided informed consent. The flowchart of the study participants is shown in Fig. 1.

### CCTA protocol

The CCTA imaging was performed via prospective electrocardiographic (ECG) gating using 256-slice multi-detector CT (Brilliance iCT; Philips Healthcare, OH, USA). The CT data acquisition was initiated under the full inspiration of 6 s after a predetermined signal attenuation threshold of 180 Hounsfield units was attained. About 60–70 mL of contrast media (Iodixanol 320; Jiangsu Hengrui Pharmaceuticals, Lianyungang, Jiangsu, China), followed by 20 mL of saline, was intravenously injected into the antecubital vein at a flow rate of 5–6 mL/s. No beta-blockers, nitroglycerin, or contrast agents were used. The imaging parameters were as follows: tube potential, 120 kVp; effective tube current–time product, 50 mA; detector configuration, 32 × 0.625 mm^2^; rotation time, 330 ms; field of view, 250 mm; reconstructed slice thickness, 2.5 mm; and an increment of 2.5 mm.

### CMRA protocol

All MRI examinations were performed using a clinical 3 T MR scanner (Ingenia CX; Philips Healthcare, Amsterdam, the Netherlands) with a 32-channel body phased-array surface coil. No beta-blockers, nitroglycerin, and contrast agents were used. After a free-breathing, four-chamber cine imaging was acquired, the optimal data acquisition window was determined by the minimal motion phase of the right coronary ostium. A 3D whole-heart turbo-field gradient-echo sequence was used with ECG-gating and diaphragm navigator-gating for NCMRA data acquisition.

All patients underwent four different scanning sequences in a random order: the conventional SENSE sequence (denoted as SENSE), and the other three sequences using CS technology with acceleration factors 4, 5, and 6 (indicated as CS4, CS5, and CS6). The detailed imaging parameters considered in this study are listed in Table 1. The SENSE sequence was considered the control sequence, and CCTA images were taken as the gold standard.

**Table 1:** Image parameters of the 4 sequences of NCMRA

Sequence type	SENSE	CS4	CS5	CS6
FOV (mm)	350 × 350	350 × 350	350 × 350	350 × 350
Slice thickness (mm)	1.5	1.5	1.5	1.5
Slice number	140	140	140	140
Reconstruction voxel size (mm)	0.63 × 0.63 × 0.75	0.63 × 0.63 × 0.75	0.63 × 0.63 × 0.75	0.63 × 0.63 × 0.75
Acceleration factors	SENSE(2)	CS (4)	CS (5)	CS (6)
TR (ms)	3.3	3.3	3.3	3.3
TE (ms)	1.3	1.3	1.3	1.3
Flip angle (^0^)	17	17	17	17
Scan time (s)	343.27 ± 44.57	269.91 ± 34.82	214.97 ± 28.31	190.19 ± 21.41

### Vessel length analysis

**Fig. 2:**
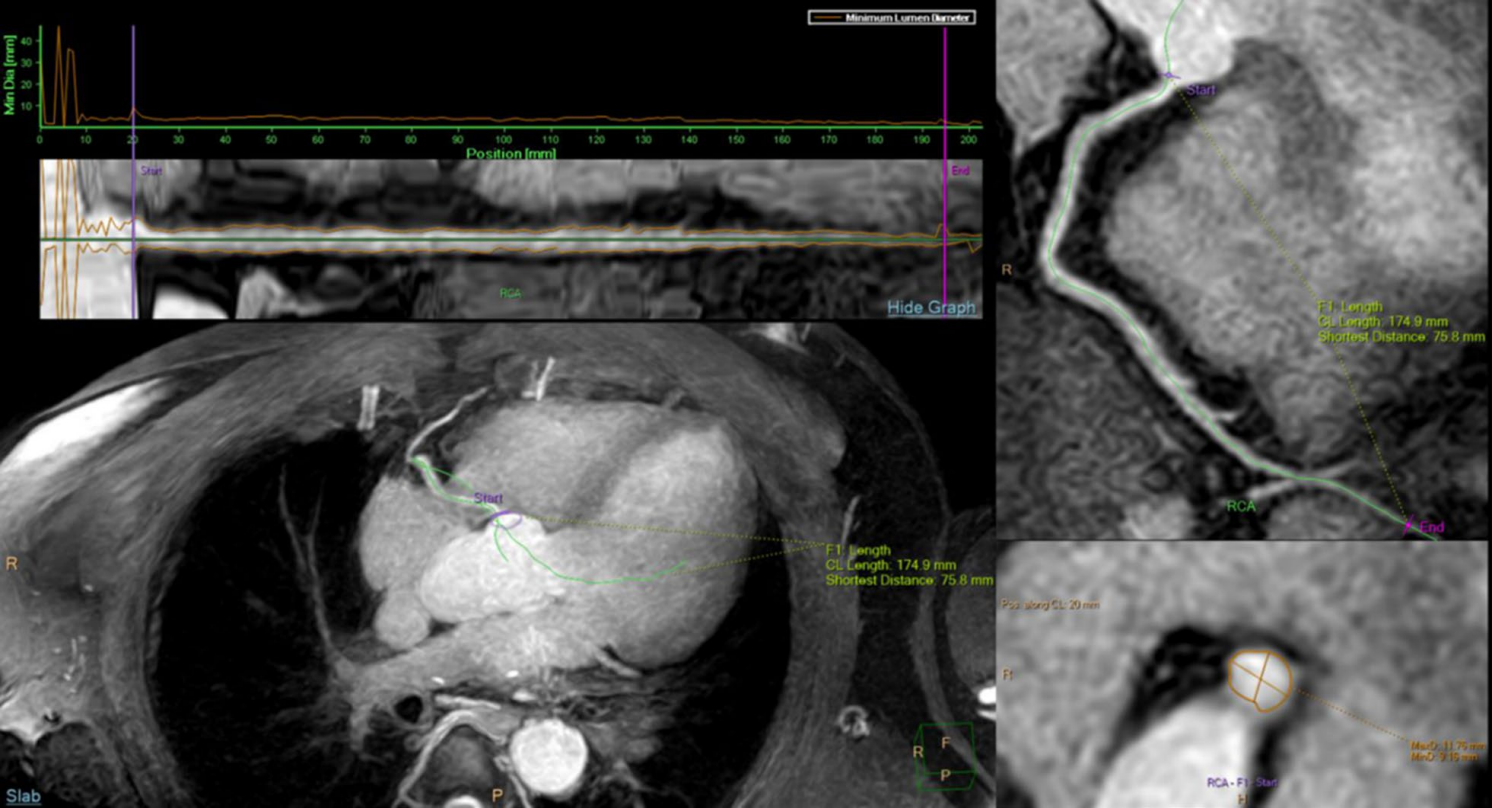
Sample coronary artery CPR and visible length measurement using IntelliSpace Portal workstation. CPR, curved planar reconstruction

All original-source CT and MR images were transferred to the IntelliSpace Portal, Version 7.0 (Philips Healthcare) for curved planar reconstruction (CPR) and vessel length measurement (Fig. 2). The visible vessel lengths measured using NCMRA sequences were compared with those measured using CCTA. Two radiologists with 8 years (Y.Z.) and 3 years (X.H.) of experience in CMRA performed CPR blindly on each set of CCTA and NCMRA images. Then, the visualized vessel lengths of the right coronary artery (RCA), left anterior descending artery (LAD; including the left main artery), and left circumflex artery (LCX) were obtained from the CPR images semiautomatically in the workstation.

The intra- and inter-observer reliabilities of visible lengths were assessed in 50 coronary arteries randomly selected using Bland–Altman plots. The intra-observer reliability was derived from repeated measurement by one radiologist (Y.Z.) after at least 1 week of blinding to the previous results. The inter-observer reliability was independently assessed by both radiologists (Y.Z. and X.H.).

### Image quality evaluation

**Fig. 3:**
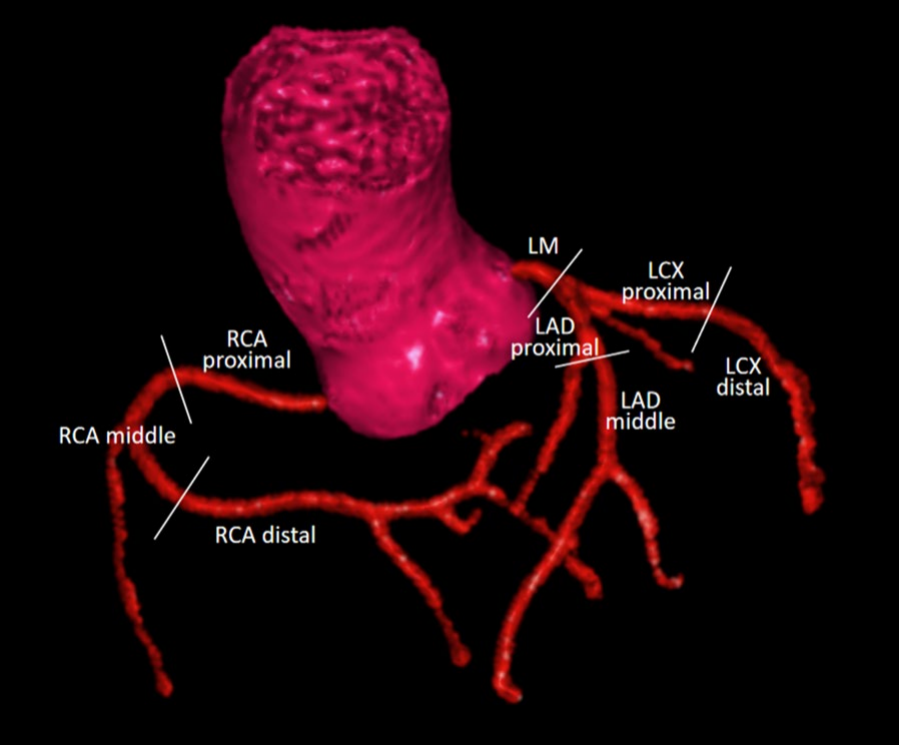
Eight coronary artery segments evaluated in this study

According to the 15-segment American Heart Association classification [28], we evaluated three segments (proximal, middle, and distal) of RCA, left main artery (LM), two segments (proximal and middle) of LAD, and two segments (proximal and distal) of LCX (Fig. 3).

Two other experienced radiologists (B.P. and Y.Y.) with more than 5 years of experience in CMRA, who were blinded to the CCTA results, determined the subjective image scores and CR independently.

#### Image quality scores

**Fig. 4:**
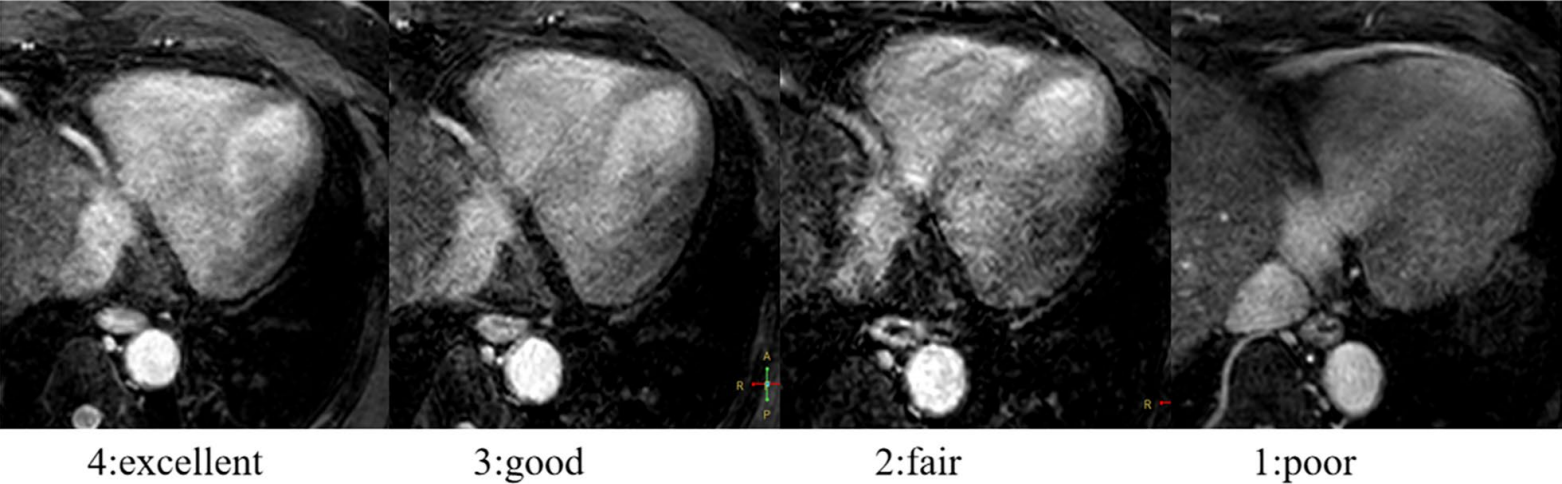
Four-point subjective score for the qualitative image analysis

A four-point subjective score was used to assess the quality of the NCMRA image: 4, excellent (vessels that were well depicted with sharply defined borders); 3, good (vessels that were adequately visualized with only mildly blurred boundaries); 2, fair (coronary vessels that were visible, but with low confidence in the diagnosis due to moderately blurred borders); and 1, poor (coronary vessels that were barely seen or obscured by noise) [29] (Fig. 4).

#### Contrast ratio

It would be inaccurate to use the classic measurement approaches for calculating the SNR and CNR because the iterative reconstruction of CS could result in an artificial reduction in noise in MR images [30]. Instead, the CRs among the eight coronary artery segments and myocardium were calculated as ${\text{CR}} = \frac{{\left| {\mu_{{{\text{vessel}}}} - \mu_{{{\text{myocardium}}}} } \right|}}{{\sqrt {\sigma_{{{\text{vessel}}}}^{2} + \sigma_{{{\text{myocardium}}}}^{2} } }}$, where *μ* is the mean signal intensity of the corresponding tissue and *σ* is the variance of the related tissue [31]. The regions of interest (ROIs) were placed on the same slices of the four NCMRA source images for measuring the signal intensity of the arteries for each segment, while ROIs of the myocardium were drawn on the left ventricular septum.

The intra- and inter-observer reliabilities of the image scores and CR were assessed in 50 segments randomly selected using the Kappa test and Bland–Altman plots, respectively. The intra-observer reliability was derived from repeated measurement by one radiologist (Y.Z.) after at least 1 week of blinding to the previous results. Two radiologists independently assessed the inter-observer reliability (B.P. and Y.Y.).

### Statistical analysis

The data from participants with successful NCMRA and CCTA were statistically analyzed using SPSS Statistics 26.0 (SPSS, Inc., IL, USA). The Shapiro–Wilk test was used to assess the normal distribution of the continuous data. The quantitative variables were reported as mean ± standard deviation if normally distributed and as a median or interquartile range in case of non-normal distribution, and categorical variables were expressed as numbers (percentage).

The differences between the scan times of NCMRA sequences were assessed using the Friedman nonparametric statistical test. The linear regression analyses with the coefficient of determination (*R*^2^) were performed to determine the correlation between the visible vessel lengths of four NCMRA and CCTA sequences. A further agreement was tested via the Bland–Altman analysis (mean difference and upper and lower limits of agreement). The Friedman tests were used to compare image quality scores and CR among these sequences. Multiple comparisons were performed between three CS factor sequences and the SENSE sequences using Wilcoxon signed-rank tests. A *p* value < 0.05 indicated a statistically significant difference.

## Results

### Participant cohorts

Forty-seven participants were recruited for this study. Thirty-nine patients (83%) underwent whole-heart NCMRA, and eight patients were excluded based on the exclusion criteria. Subsequently, 36 patients (76.6%) successfully completed the CCTA and 4 NCMRA sequences (18 men and 18 women). The age of the participants ranged from 35 to 73 (mean, 52.19 ± 9.96) years, and the body mass index ranged from 16.61 to 30.92 (mean, 24.79 ± 3.41) kg/m^2^.

The Friedman test results showed that the difference between the effective scan time of the SENSE sequence (328 s (315.5–359.5 s)), and CS4 (264 s (248–284 s)), CS5 (205 s (197–227 s)), and CS6 sequence (187 s (178.5–193 s)) was statistically significant (χ^2^ = 108.773, *p* < 0.001).

### Vessel length analysis

**Fig. 5:**
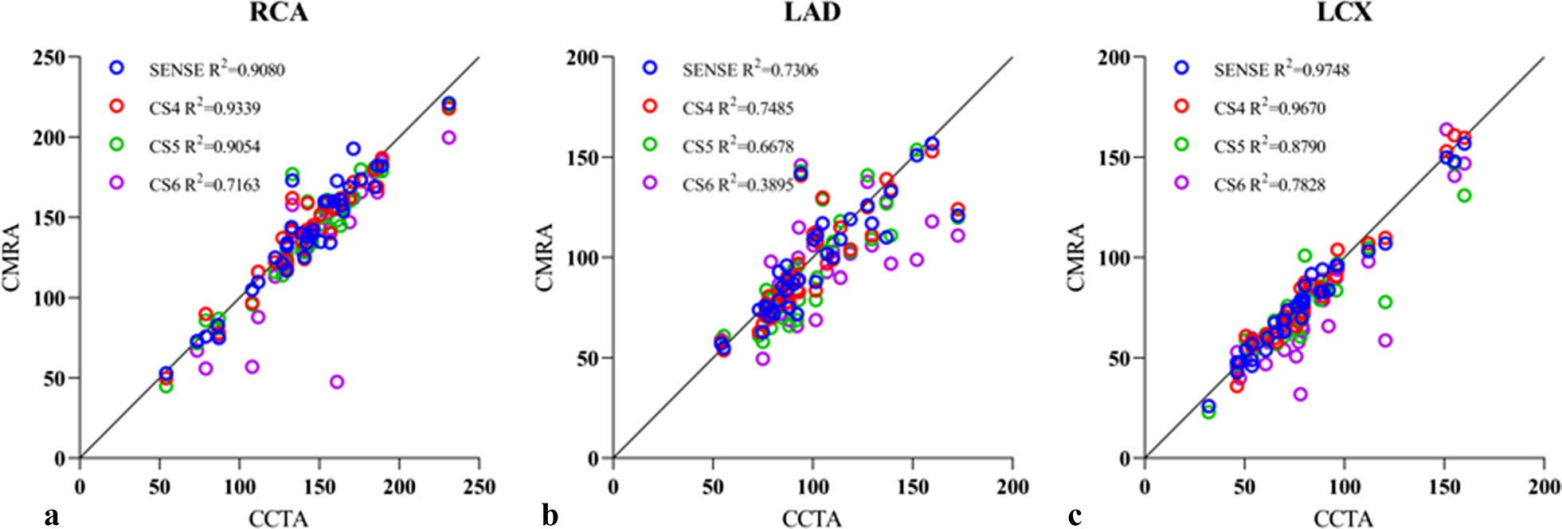
Scatter plots for vessel measurements using CCTA and NCMRA with four acceleration factors and three arteries. **a** Right coronary artery, **b** left anterior descending artery, and **c** left circumflex artery

A linear regression analysis revealed that the visible length of RCA and LCX measured via SENSE and CS4 sequences correlated well with that of the CCTA sequence (*R*^2^ > 0.90). It was because the correlation decreased gradually with the increase in the acceleration factor (*R*^2^, 0.72–0.91). The vessel length of LAD measured by each NCMRA sequence was slightly less correlated with that measured via the CCTA sequence (*R*^2^, 0.39–0.73) (Fig. 5).

**Fig. 6:**
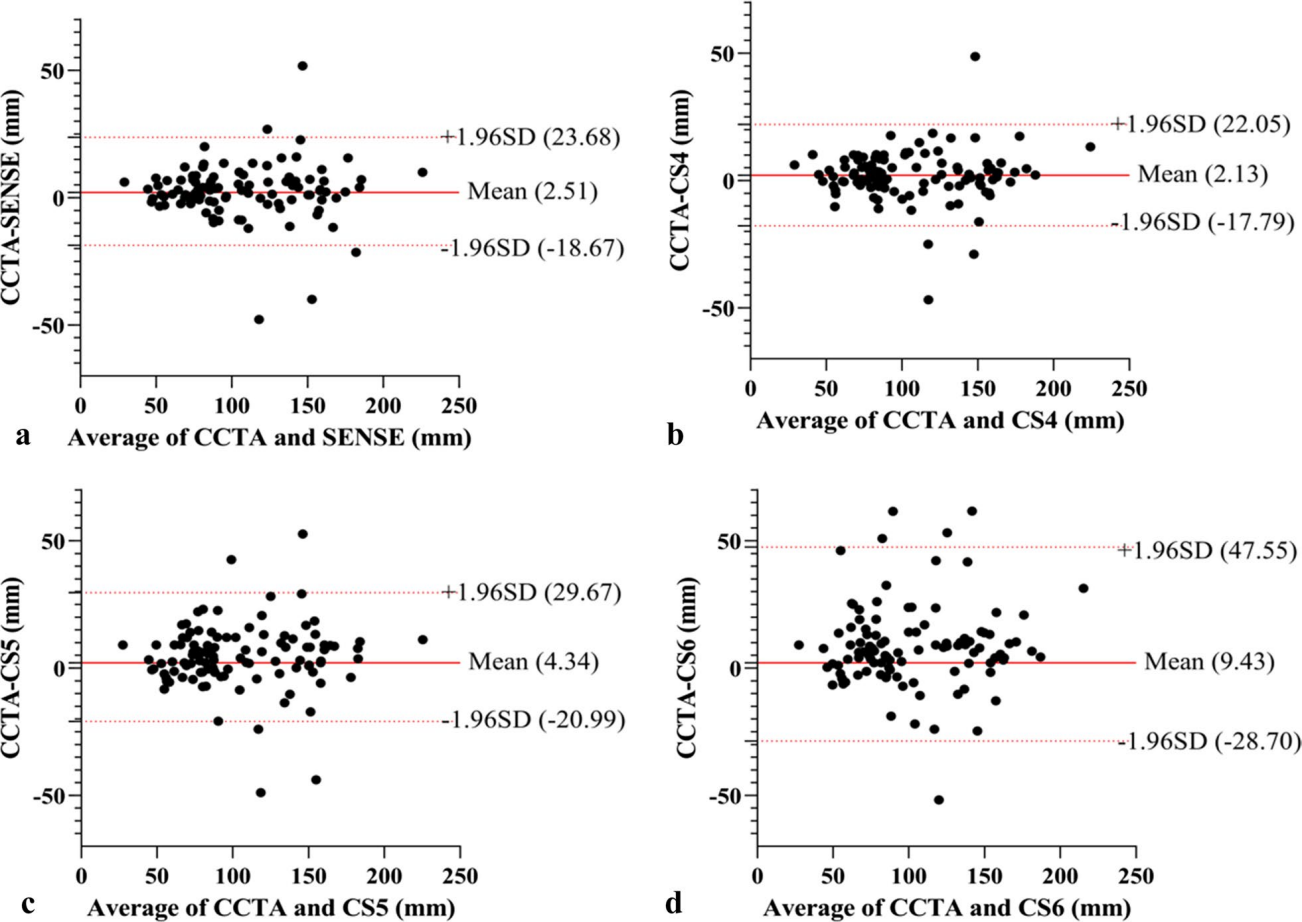
Bland–Altman analysis for visible vessel lengths compared between CCTA and NCMRA sequences. **a** CCTA versus SENSE, **b** CCTA versus CS4, **c **CCTA versus CS5, and **d** CCTA versus CS6. The red solid line indicates the mean difference between CCTA and CMRA sequences; the red dashed lines indicate the 95% limits of the agreement interval

The Bland–Altman analysis indicated the following mean differences and limits of agreement (LOAs) in visible vessel length measurements between CCTA and NCMRA sequences: 2.51 mm (95% LOA; − 18.67 to 23.68 mm) between CCTA and SENSE sequence; 2.13 mm (95% LOA; − 17.79 to 22.05 mm) between CCTA and CS4 sequence; 4.34 mm (95% LOA; − 20.99 to 29.67 mm) between CCTA and CS5 sequence; and 9.43 mm (95% LOA; − 28.7 to 47.55 mm) between CCTA and CS6 sequence. A detailed overview, scatter plots, and Bland–Altman plots of representative parameters are presented in Table 2 and Fig. 6.

**Table 2:** Visible length of the coronary artery of CCTA and NCMRA

	Visible length of CCTA (mm)	SENSE		CS4		CS5		CS6	
		Visible length (mm)	*R*^2^	Visible length (mm)	*R*^2^	Visible length (mm)	*R*^2^	Visible length (mm)	*R*^2^
RCA	144 [127.625–163.8]	139.35 [121.9–166.9]	0.908	143.9 [122.15–161.875]	0.9339	142.85 [118.625–159.875]	0.9054	139.3 [114.625–157.775]	0.7163
LAD	92.3 [81.175–112.85]	88.95 [74.9–115.375]	0.7306	88.35 [77.15–114.15]	0.7485	88.35 [71.65–110.4]	0.6678	91.4 [72.15–105.9]	0.3895
LCX	77.5 [60.8–89.3]	75.85 [57.675–90.275]	0.9748	73.15 [60.075–87.25]	0.967	70.35 [58.9–83.9]	0.879	64.4 [54.4–83.375]	0.7828

Metric data are reported as median and interquartile range

*RCA* right coronary artery; *LAD* left anterior descending artery; *LCX* left circumflex artery

**Fig. 7:**
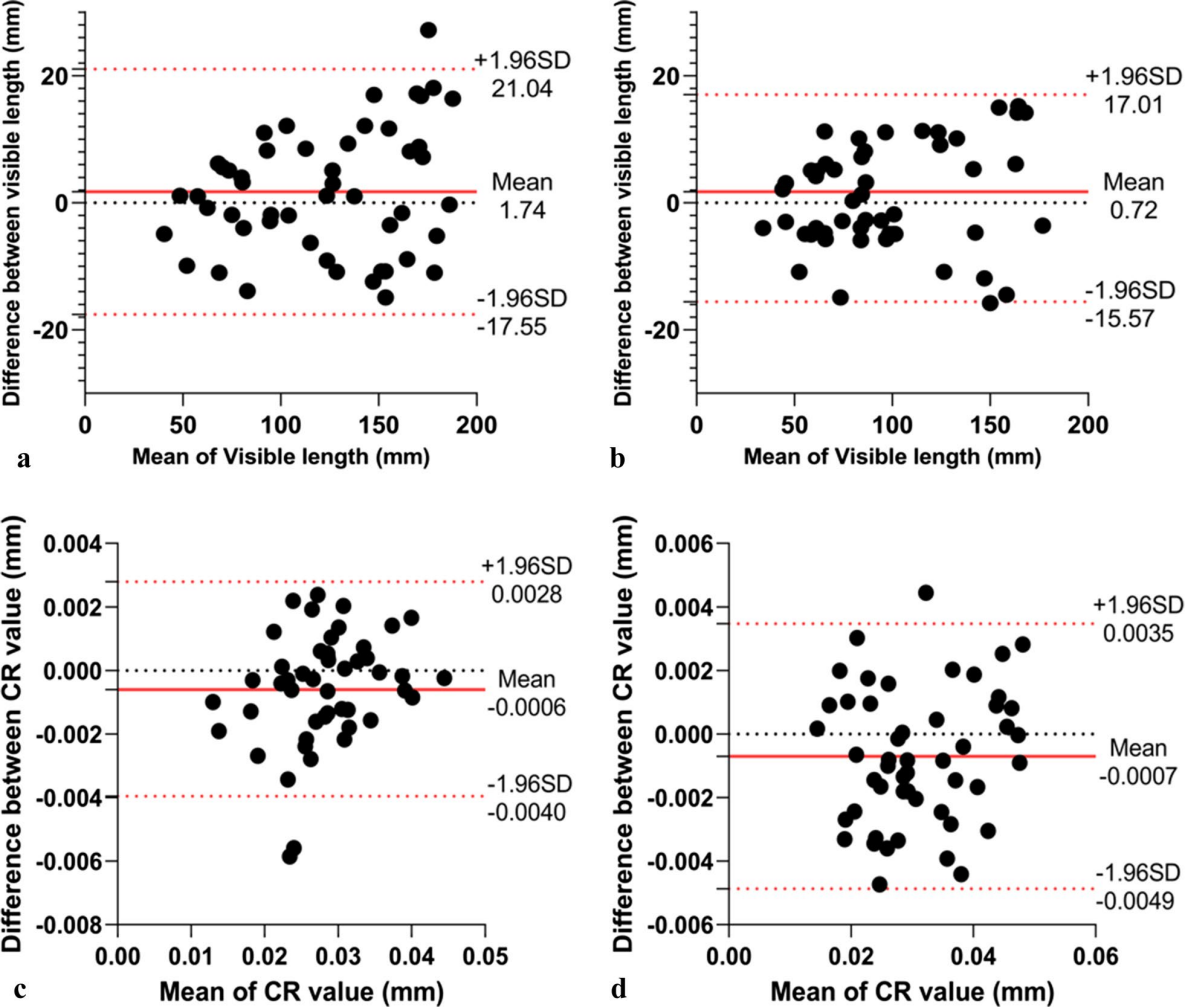
**a**, **b** Bland–Altman analysis of intra- (**a**) and inter-technique (**b**) reproducibility of visible vessel lengths. **c**, **d** Bland–Altman analysis of intra- (**c**) and inter-technique (**d**) reproducibility of CR. The solid red line indicates the mean difference; the red dashed lines indicate the 95% limits of the agreement interval

In randomly selected 50 blood vessels, the visible length had intra-observer reliability of 1.74 ± 19.3 mm and inter-observer reliability of 0.72 ± 16.29 mm (Fig. 7a, b).

### Image quality scores

**Fig. 8:**
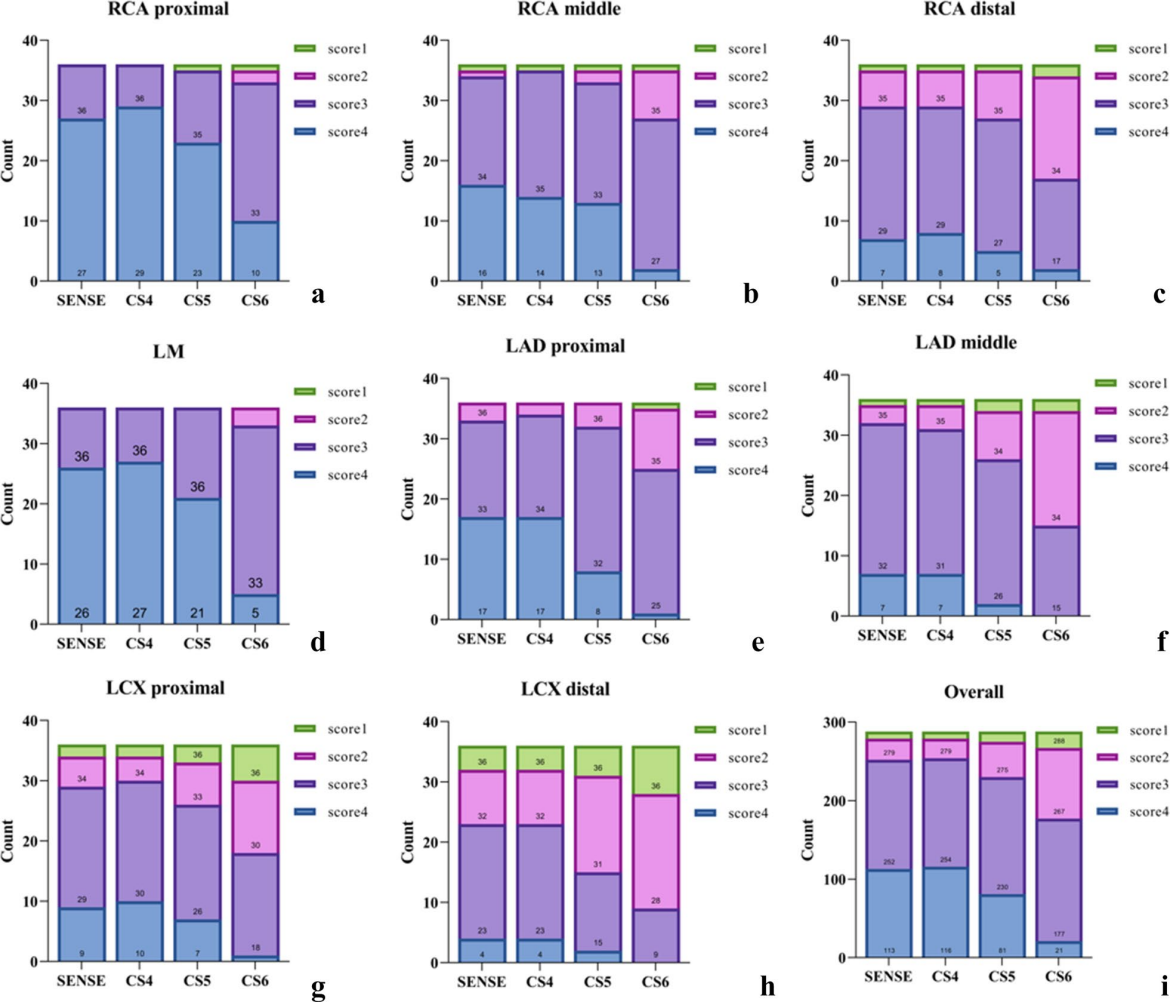
Stacked graphs of coronary artery segments (**a**–**h**) and overall image quality scores (**i**). No significant differences were observed between SENSE and CS4 sequences (*p* > 0.05)

A total of 1152 coronary segments from the 4 NCMRA sequences of 36 participants were analyzed, of which 331 coronary segments (28.7%) were analyzed subjectively with a score of 4, 582 segments (50.5%) with a score of 3, 187 segments (16.2%) with a score of 2, and 52 segments (4.5%) with a score of 1 (Fig. 8).

**Fig. 9:**
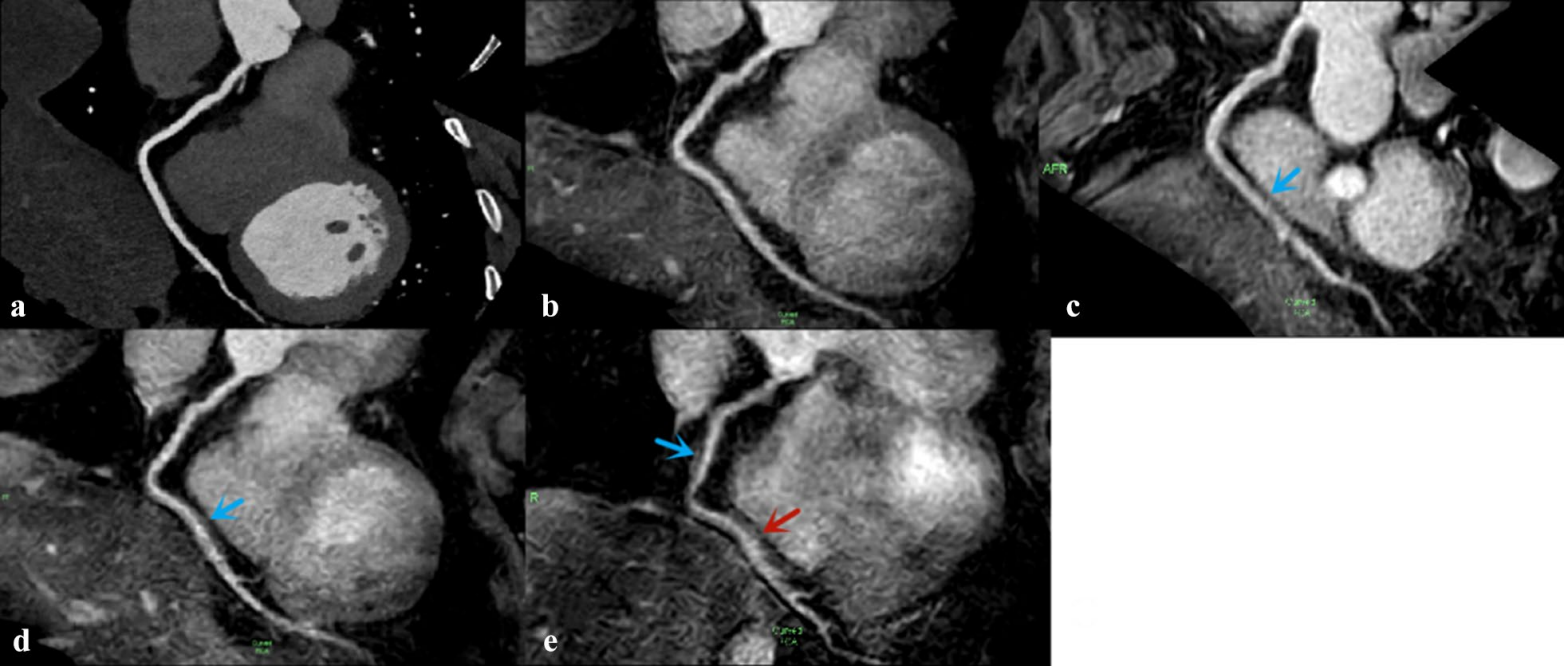
RCA image of a 41-year-old man acquired via CCTA sequence (**a**) and four NCMRA sequences. On the image from SENSE sequence (**b**), the blood vessels were well depicted with sharply defined borders, and the score was 4. The distal segments of RCA from CS4 sequence (**c**) and CS5 sequence (**d**) were adequately visualized with only mildly blurred borders, and the score was 3 (*blue arrow*). On the image from CS6 sequence (**e**), the proximal and middle segments of RCA were adequately visualized (*blue arrow*), but the distal part was visible with moderately blurred borders; the score was 2 (*red arrow*)

No significant differences in the image scores were observed between SENSE and CS4 sequences (*p* > 0.05). The image scores of LAD and LCX from CS5 sequence, and all segments from CS6 sequence were lower than those from the CS4 sequence (*p* < 0.05) (Tables 3 and 4 and Fig. 9).

**Table 3:** Average image scores of coronary segments in NCMRA

	Average image score					*P*	CS4 Versus SENSE		CS5 Versus SENSE		CS6 Versus SENSE		CS4 Versus CS5		CS4 Versus CS6		CS5 Versus CS6	
	SENSE	CS4	CS5	CS6	*x*^2^		*Z*	*P*	*Z*	*P*	*Z*	*P*	*Z*	*P*	*Z*	*P*	*Z*	*P*
RCApro	4 [3.25–4]	4 [4–4]	4 [3–4]	3 [3–4]	46.673	< 0.001*	1.414	0.157	1.897	0.058	4.2	< 0.001*	2.53	0.011*	4.413	< 0.001*	3.873	< 0.001*
RCAmid	3 [3–4]	3 [3–4]	3 [3–4]	3 [2.25–3]	38.533	< 0.001*	0.378	0.795	1.265	0.206	4.583	< 0.001*	1	0.317	4.264	< 0.001*	3.71	< 0.001*
RCAdis	3 [3–3]	3 [3–3]	3 [2.25–3]	2 [2–3]	22.635	< 0.001*	0.302	0.763	1.069	0.285	3.079	0.002*	1.89	0.059	3.508	< 0.001*	2.977	0.003*
LM	4 [3–4]	4 [3.25–4]	4 [3–4]	3 [3–3]	47.765	< 0.001*	0.333	0.739	1.387	0.166	4.707	< 0.001*	1.897	0.058	5	< 0.001*	4.359	< 0.001*
LADpro	3 [3–4]	3 [3–4]	3 [3–3]	3 [2–3]	39.483	< 0.001*	0.277	0.782	2.134	0.033*	4.044	< 0.001*	2.4	0.016*	4.735	< 0.001*	3.419	0.001*
LADmid	3 [3–3]	3 [3–3]	3 [2–3]	2 [2–3]	46.639	< 0.001*	0.447	0.655	3	0.003*	4.327	< 0.001*	3.051	0.002*	4.536	< 0.001*	3.606	< 0.001*
LCXpro	3 [3–3.75]	3 [3–4]	3 [2–3]	2.5 [2–3]	36.815	< 0.001*	0.816	0.414	1.604	0.109	4.185	< 0.001*	2	0.046*	4.413	< 0.001*	3.532	< 0.001*
LCXdis	3 [2–3]	3 [2–3]	2 [2–3]	2 [1–2]	33.103	< 0.001*	0	1	2.524	0.012*	4.184	< 0.001*	2.84	0.005*	3.841	< 0.001*	2.517	0.012*
All	3 [3–4]	3 [3–4]	3 [3–4]	3 [2–3]	302.348	< 0.001*	0.63	0.529	5.086	< 0.001	11.641	< 0.001*	6.263	< 0.001*	12.22	< 0.001*	9.823	< 0.001*

Metric data are reported as median and interquartile range

*RCA* right coronary artery; *LAD* left anterior descending artery; *LCX* left circumflex artery; *pro* proximal; *mid* middle; *dis* distal

*The difference was statistically significant (*p* < 0.05)

**Table 4:** Average image scores of coronary arteries in NCMRA

	Average image score				CS4 Versus SENSE			CS5 Versus SENSE		CS6 Versus SENSE		CS4 Versus CS5		CS4 Versus CS6		CS5 Versus CS6	
	SENSE	CS4	CS5	CS6	*Z*	*P*		*Z*	*P*	*Z*	*P*	*Z*	*P*	*Z*	*P*	*Z*	*P*
RCA	3 [3–4]	3 [3–4]	3 [3–4]	3 [2–3]	0.447	0.655		2.403	0.016*	6.689	< 0.001*	3.138	0.002*	6.997	< 0.001*	6.037	< 0.001*
LAD	3 [3–4]	3 [3–4]	3 [3–4]	3 [2–3]	0.192	0.847		3.779	< 0.001*	7.503	< 0.001*	4.221	< 0.001*	8.225	< 0.001*	6.576	< 0.001*
LCX	3 [2–3]	3 [2–3]	3 [2–3]	2 [2–3]	0.5	0.617		2.959	0.003*	5.905	< 0.001*	3.413	0.001*	5.798	< 0.001*	4.297	< 0.001*

Metric data are reported as median and interquartile range. LAD including the left main artery

*RCA* right coronary artery; *LAD* left anterior descending artery; *LCX* left circumflex artery

*The difference was statistically significant (*p* < 0.05)

Furthermore, of all coronary segments with score 4 from NCMRA sequences, 71.9% (238/331) were located in the proximal segments of the LM and 70.3% (168/239) segments with scores 1 and 2 were located in the LAD middle, RCA distal, and LCX distal segments.

Good intra-observer (kappa score = 0.642) and inter-observer agreements (kappa score = 0.613) were observed with respect to the image quality of NCMRA.

### CR analysis

**Fig. 10:**
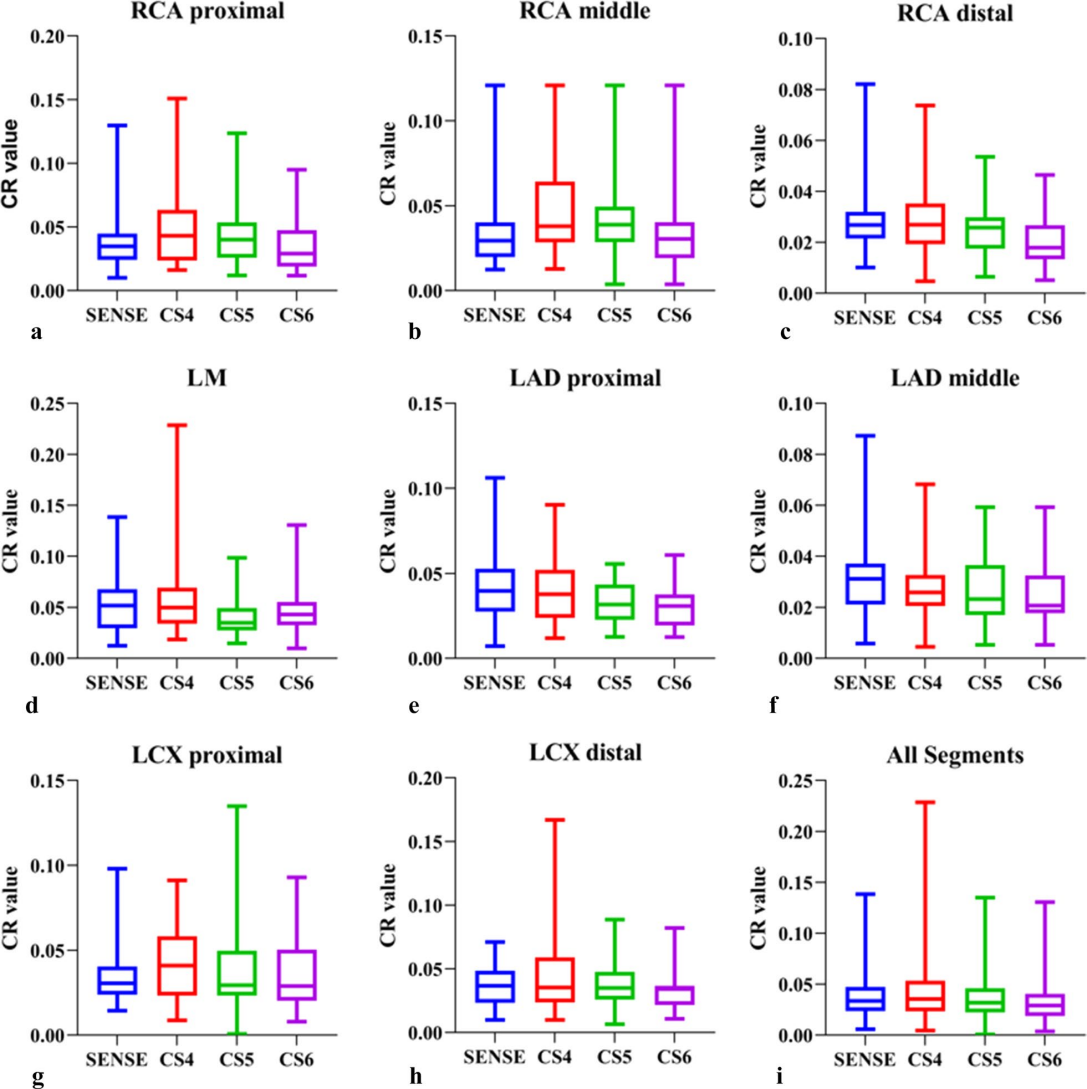
The CR values of coronary artery segments (**a–h**) and overall artery segments (**i**). In comparison to the SENSE sequence, the CR values for the RCA pro, the RCA mid, and the overall segments were higher in the CS4 sequence. From the CS6 sequence, the mean CR was lower than from the SENSE sequence. The CS5 and SENSE sequences did not show any statistically significant differences

The CR of each vessel segment was calculated (Table 5 and Fig. 10). The mean CR decreased slightly and gradually for most arteries with the increase in the CS acceleration factor. The mean CR from the CS4 sequence was higher than that from the SENSE sequence (*Z* = 2.255,* p* = 0.024). The mean CR from the CS6 sequence was lower than that from the SENSE sequence (*Z* = 5.467,* p* < 0.001). No statistically significant differences in CR were observed between CS5 and SENSE sequences (*p* > 0.05).

**Table 5:** CR values of coronary segments in NCMRA

	CR value				× 2	*P*	CS4 Versus SENSE		CS5 Versus SENSE		CS6 Versus SENSE		CS4 Versus CS5			CS4 Versus CS6			CS5 Versus CS6	
	SENSE	CS4	CS5	CS6			*Z*	*P*	*Z*	*P*	*Z*	*P*	*Z*	*P*		*Z*	*P*		*Z*	*P*
RCA pro	0.0348[0.0232–0.0448]	0.0431[0.0237–0.0634]	0.0400[0.0259–0.0536]	0.0291[0.0189–0.0474]	13.588	0.004*	2.451	0.014*	1.194	0.232	1.257	0.209	1.493	0.136		3.374	0.001*		2.522	0.012*
RCA mid	0.0294[0.0197–0.0402]	0.0380[0.0284–0.0643]	0.0388[0.0285–0.0495]	0.0304[0.0191–0.0404]	12.437	0.006*	3.018	0.003*	1.154	0.248	1.065	0.287	1.778	0.075		3.12	0.002*		2.461	0.014*
RCA dis	0.0268[0.0215–0.0319]	0.0269[0.0193–0.0354]	0.0258[0.0175–0.0298]	0.0179[0.0133–0.0267]	19.149	< 0.001*	0.344	0.731	1.097	0.272	3.407	0.001	1.462	0.144		3.922	< 0.001*		2.783	0.005*
LM	0.0518[0.0295–0.0678]	0.0498[0.0339–0.0694]	0.0431[0.0324–0.0555]	0.0348[0.0275–0.0493]	15.353	0.002*	0.628	0.53	1.084	0.278	2.121	0.034*	2.529	0.011*		3.362	0.001*		1.438	0.15
LAD pro	0.0397[0.0275–0.0528]	0.0378[0.0239–0.0522]	0.0317[0.0227–0.0435]	0.0308[0.0193–0.0376]	22.875	< 0.001*	1.159	0.246	2.248	0.025*	3.479	0.001*	1.744	0.081		3.253	0.001*		2.058	0.040*
LAD mid	0.0312[0.0210–0.0372]	0.0256[0.0206–0.0328]	0.0233[0.0170–0.0367]	0.0207[0.0178–0.0326]	17.803	< 0.001*	3.19	0.001*	2.099	0.036*	2.932	0.003*	0.934	0.35		2.13	0.033*		1.823	0.068
LCX pro	0.0306[0.0239–0.0405]	0.0409[0.0233–0.0582]	0.035[0.0259–0.0477]	0.0341[0.0216–0.0367]	6.279	0.099	1.735	0.083	0.896	0.371	0.943	0.346	0.408	0.683		1.807	0.071		2.679	0.007*
LCX dis	0.0367 [0.0233–0.0486]	0.0352[0.0236–0.0591]	0.0295[0.0233–0.0498]	0.0289[0.0202–0.0503]	2.65	0.449	1.481	0.138	0.848	0.396	0.801	0.423	1.147	0.251		1.178	0.239		1.308	0.191
All	0.0336[0.0236–0.0474]	0.0354[0.0233–0.0536]	0.0325[0.0222–0.0476]	0.0292[0.0191–0.0399]	78.58	< 0.001*	2.255	0.024*	1.453	0.146	5.467	< 0.001*	3.901	< 0.001*		7.462	< 0.001*		6.055	< 0.001*

Metric data are reported as median and interquartile range

*RCA* right coronary artery; *LAD* left anterior descending artery; *LCX* left circumflex artery; *pro* proximal; *mid* middle; *dis* distal

*The difference was statistically significant (*p* < 0.05)

In randomly selected 50 segments, CR had intra-observer reliability of − 0.0006 ± 0.0033 and inter-observer reliability of − 0.0007 ± 0.0042 (Fig. 7c, d).

## Discussion

In this study, the vendor-provided CS tool with three different acceleration factors was used for NCMRA. A reference CCTA technique and a sequence using the conventional SENSE technique were included for comparative analysis. We observed that the CMRA CS4 sequence could provide a balance between the image quality and acquisition time compared with the longer acquisition time of NCMRA SENSE. When the CS acceleration factors increased from 4 to 6, the scanning time decreased gradually and the image quality decreased subtly.

The mean total MR scan time (including scout imaging) for imaging of the coronary artery in a patient was 70 min in 2001 [32]. Although MRI technology has improved significantly in the last two decades, the scanning time for CMRA is still extremely long, and its clinical application is limited. In this study, the effective acquisition time for the conventional SENSE sequence was 343 s, while the effective acquisition time for CS4, CS5, and CS6 was 269 s, 215 s, and 190 s, indicating a decrease in the acquisition time by 21.4%, 37.4%, and 44.6%, respectively. The scan time for the CS5 sequence in this study was similar to that in the study by Nakamura and Hirai, while the scan time for CS4 was slightly longer and the scan time for CS6 was slightly shorter [26, 33]. The acquisition times for CS NCMRA observed in this study were short enough for acquisition in the waiting period between contrast injection and late gadolinium enhancement (LGE) imaging. The contrast agents could shorten the T1 relaxation time of the coronary artery blood pool and help improve SNR and image quality. The contrast-enhanced CS CMRA (CE-CMRA) could further shorten the acquisition time considerably while maintaining the image quality compared with the NCMRA [27, 33]. In the study by Ogawa et al. [34], CMRA with a CS factor of 7.6 was completed within the waiting time of the LGE CMR. The mean acquisition time of 207 s was obtained, and the results indicated that CE-CMRA could detect significant stenoses with comparable sensitivity and specificity. We hypothesized that integrating 3D whole-heart CMRA into the protocols for myocardial perfusion or LGE would improve CAD accuracy even more.

In this study, when the CS factor increased, the image quality scores and CR reduced possibly because of the sparse data sampling achieved using higher acceleration factors for scanning [20]. CS4 could generate images with almost equally acceptable quality compared with the SENSE sequence but with a reduction of 21.4% in scan time. Furthermore, the lengths of the three coronary arteries measured using SENSE and CS4 sequences were all well correlated with the vessel lengths measured using CCTA (*R*^2^ of the three vessels between SENSE and CCTA was 0.91, 0.73, and 0.97, and that between CS4 and CCTA was 0.93, 0.75, and 0.97, respectively). As the CS acceleration factor increased from 4 to 5, the scan time was reduced by 37.4%. Although the CR decreased slightly, the overall CR was not statistically significantly different in the CS4 sequence compared with that in the SENSE sequence (*p* > 0.05). However, the overall image score of the CS4 sequence was significantly lower than that of the SENSE sequence (*p* < 0.001). As the acceleration factor increased to 6, the image artifacts became obvious and the boundaries of the arteries became rougher. Both CR and image quality score were significantly lower in the CS4 sequence than in the SENSE sequence (*P* < 0.001).

The mean image scores and CR of proximal vessels were higher than those of middle and distal vessels. This might be related to the thin vessels in the distal segments, which could not generate sufficient signal intensity. Similarly, in the study by Hajhosseiny et al. [35], 63% (15/24) of nondiagnostic CMRA segments were located in the distal segments. Nakamura et al. performed CMRA using CS factor 9 and found that the lesions at the peripheral small vasculatures were blurred on CS CMRA images [26].

The correlation of the visible vessel length between CMRA and CCTA techniques for LAD was lower than that of RCA and LCX because of the following reasons: (1) The right coronary dominance was the most common type of coronary circulation, and the distal branches of LAD and LCX were thinner and could not generate sufficient signal intensity. Using contrast agents or sublingual nitroglycerin might improve the visualization of the vessels [36]. (2) A certain difference existed between the optimal systolic acquisition windows for LAD and RCA in some patients. In our study, the acquisition window was set to end-systolic and early-diastolic intervals in patients with high heart rates (> 70 bpm); the mid-diastolic intervals were used to obtain images in patients with low heart rates (< 70 bpm). The motion of LAD further increased the image artifacts when the systolic minimal motion phase of the right coronary was selected as the optimal acquisition window. Moreover, the motion difference was almost nonexistent when the acquisition window was set to the end of diastole. Furthermore, Seifarth et al. found the least variability between the optimal reconstruction intervals of the two vessels in patients presenting with a heart rate between 60 and 80 bpm [37]. Therefore, it was possible to achieve better image quality when using beta-blockers to control the heart rate at 60–80 bpm.

This study had several limitations. First, this study was performed in only one hospital involving a small number of participants. Second, we only included patients with suspected CAD, and the intermediate-to-high-risk patients were not evaluated in this study. Further studies involving a larger group of participants should be carried out for evaluating the efficiency and clinical use of CS CMRA. Third, CMRA sequences were acquired without a contrast medium. The CS factor of 4 does not represent the optimal agreement between image quality and acquisition time while scanning with a contrast medium. Finally, we did not perform stenotic vessel evaluations. Previous studies of contrast-enhanced conventional CMRA and NCMRA using a 3-T scanner showed the detection of significant CAD with good diagnostic performance [12]. Further studies are needed to reveal the accuracy of CS CMRA in assessing obstructive CAD.

## Conclusions

NCMRA techniques using the CS sequences could considerably shorten the acquisition time. The CS sequence with an acceleration factor of 4 was generally acceptable for NCMRA in clinical situations using a 3-T scanner to balance the image quality and acquisition time.

## Data Availability

The original contributions presented in the study are included in the article/supplementary material; further inquiries can be directed to the corresponding authors.

## Abbreviations

CAD: Coronary artery disease
CAG: Coronary X-ray angiography
CCTA: Coronary CT angiography
CPR: Curved planar reconstruction
CR: Contrast ratio
CS: Compressed SENSE
LAD: Left anterior descending artery
LCX: Left circumflex artery
LGE: Late gadolinium enhancement
LM: Left main artery
LOA: Limits of agreement
NCMRA: Noncontrast coronary MR angiography
RCA: Right coronary artery
SENSE: Sensitivity encoding
